# The cGAS-STING pathway in cancer immunity: dual roles, therapeutic strategies, and clinical challenges

**DOI:** 10.1042/EBC20253006

**Published:** 2025-03-07

**Authors:** Beilei Yue, Wenbo Gao, Jonathan F. Lovell, Honglin Jin, Jing Huang

**Affiliations:** 1 College of Biomedicine and Health and College of Life Science and Technology, Huazhong Agricultural University, Wuhan, 430070, China; 2 Department of Biomedical Engineering, University at Buffalo, State University of New York, Buffalo, NY 14260, U.S.A.; 3 Department of Radiation and Medical Oncology, Zhongnan Hospital of Wuhan University, Wuhan, 430071, China; 4 Hubei Province Key Laboratory of Precision Radiation Oncology, Wuhan, 430022, China

**Keywords:** cancer immunotherapy, cGAS-STING pathway, chromosomal instability, immune evasion, targeted therapies

## Abstract

The cyclic GMP-AMP synthase–stimulator of interferon genes (cGAS-STING) pathway is a crucial component of the host’s innate immunity and plays a central role in detecting cytosolic double-stranded DNA from endogenous and exogenous sources. Upon activation, cGAS synthesizes cGAMP, which binds to STING, triggering a cascade of immune responses, including the production of type I interferons and pro-inflammatory cytokines. In the context of cancers, the cGAS-STING pathway can exert dual roles: on the one hand, it promotes anti-tumor immunity by enhancing antigen presentation, stimulating T-cell responses, and inducing direct tumor cell apoptosis. On the other hand, chronic activation, particularly in tumors with chromosomal instability, can lead to immune suppression and tumor progression. Persistent cGAS-STING signaling results in the up-regulation of immune checkpoint molecules such as PD-L1, contributing to immune evasion and metastasis. Consequently, anti-tumor strategies targeting the cGAS-STING pathway have to consider the balance of immune activation and the immune tolerance caused by chronic activation. This review explores the mechanisms underlying both the anti-tumor and protumor roles of the cGAS-STING pathway, with a focus on potential therapeutic approaches, and the challenges faced in their clinical application, along with corresponding solutions.

## Introduction

**Figure 1:**
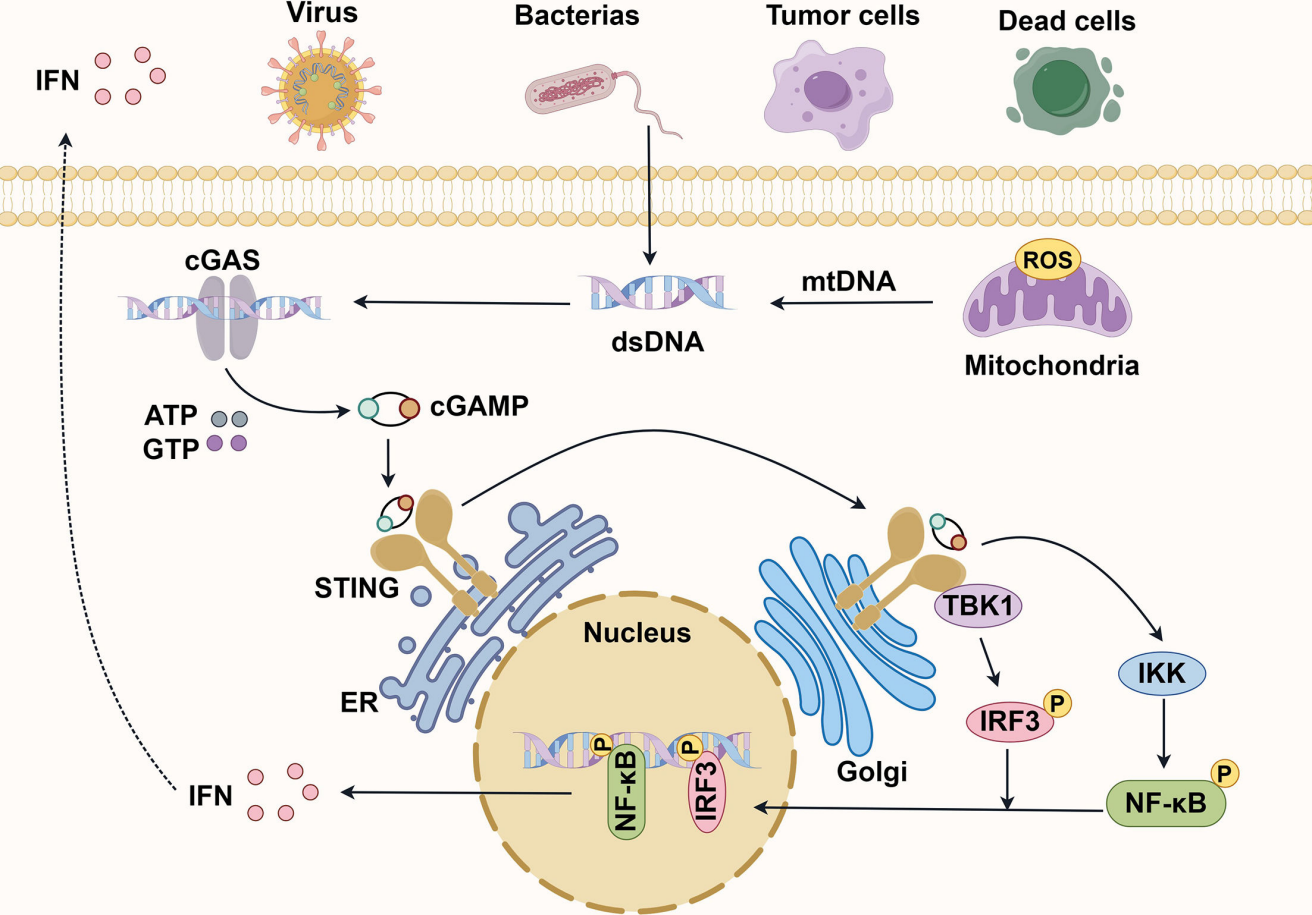
Molecular mechanisms of cGAS-STING pathway activation. cGAS recognizes both endogenous and exogenous dsDNA in a length-dependent manner. Activated cGAS employs ATP and GTP as substrates to catalyze the synthesis of the secondary messenger cGAMP. Upon binding to cGAMP, STING undergoes oligomerization to form a tetramer, subsequently trafficking to the Golgi apparatus. In the Golgi apparatus, STING undergoes palmitoylation. Then, STING recruits and activates TBK1, which catalyzes a serine residue within the pLxIS motif in STING, phosphorylates adjacent STING dimers, and activates IRF3. Phosphorylated IRF3 dimerizes and transfers to the nucleus to transcriptionally express immunostimulatory genes and IFN. Activated STING also recruits and activates inhibitor of κB kinase （IKK）. Phosphorylated IKK then phosphorylates IκB, marking it for proteasomal degradation. The degradation of IκB releases NF-κB subunits, allowing them to translocate to the nucleus, where they trigger classical NF-κB signaling and induce the production of cytokines. In conclusion, cGAS-STING activation can not only induce IFN production through IRF3/7 but also produce inflammatory cytokines such as TNF-α, IL-1β, and IL-6 by activating the NF-κB signaling pathway, which further promote the production of IFN. Created with Figdraw. cGAS, cyclic GMP-AMP synthase; cGAMP, cyclic GMP-AMP; dsDNA, double-stranded DNA; IFN, interferon; STING, stimulator of interferon genes.

Stimulator of interferon genes (STING) was identified in 2008 as a critical component of innate immunity, detecting viral infections and triggering type I interferon (IFN-I) responses [1,2]. Subsequent studies revealed cyclic GMP-AMP synthase (cGAS) as the DNA sensor that synthesizes cyclic GMP-AMP (cGAMP) to activate STING, mediating downstream signaling [3,4]. As shown in Figure 1, STING activates kinases like TANK-binding kinase 1 (TBK1), inducing IFN-I and pro-inflammatory cytokines [5].

The cGAS-STING pathway plays a crucial role in cancer therapy, not only as a therapeutic target but also as a key mediator activated during various therapeutic interventions [6,7]. Radiotherapy and chemotherapeutic agents induce DNA damage, leading to cytosolic double-stranded DNA (dsDNA) accumulation, which activates the cGAS-STING pathway to enhance tumor antigen presentation, immune recruitment, and tumor microenvironment (TME) remodeling [8–10]. Similarly, immune checkpoint blockades (ICBs), oncolytic viruses, and tumor vaccines rely on cGAS-STING to amplify immune responses, particularly T-cell activation and dendritic cell (DC) cross-presentation [11–14]. The inactivation of the cGAS-STING pathway or mutations in STING can facilitate tumor immune evasion and contribute to therapeutic resistance [15–17]. These studies underscore the importance of proper cGAS-STING activation in driving anti-tumor immune responses.

The role of the cGAS-STING in cancer immunity varies greatly across cancer types. In cancers with a high mutational burden, cGAS-STING activation enhances immune recognition, making it a potential therapeutic target [18]. However, in chromosomal instability (CIN)-driven tumors, chronic activation may promote immune suppression and tumor progression [19,20].

There have been considerable challenges in targeting the cGAS-STING pathway for cancer treatment. Clinical trials of STING agonists have reported limited efficacy due to systemic toxicity, chronic immune activation, and paradoxical tumor-promoting effects [21,22]. Chronic cGAS-STING activation can cause immune suppression through PD-L1 up-regulation and a pro-inflammatory TME that promotes tumor progression [23,24]. These complexities underscore the need for a deeper understanding of the pathway’s dual roles. In order to expand the application of cGAS-STING-related drugs in cancer treatment, researchers have combined STING agonists with other therapies to create more diverse and effective treatment options [12].

In this review, we comprehensively explore the anti-tumor and protumor aspects of the cGAS-STING pathway, discuss the challenges in its clinical application, and propose potential strategies for optimizing its clinical utility, paving the way for more effective and personalized cancer immunotherapies.

## Anti-tumor role of cGAS-STING pathway

### Anti-tumor mechanism of cGAS-STING pathway

The cGAS-STING pathway plays a pivotal role in cancer defense, particularly in tumors with CIN [5,25,26]. CIN is a hallmark of cancers and reflects chromosomal missegregation during cell division, leading to aneuploidy and genetic heterogeneity. This genomic instability is particularly relevant to the cGAS-STING pathway, as CIN-driven tumors often release dsDNA from ruptured micronuclei into the cytoplasm, which can activate the cGAS-STING pathway [27,28]. Activation of the cGAS-STING pathway results in the production of IFN-I, such as IFN-β and other pro-inflammatory cytokines. These immune mediators recruit and activate natural killer (NK) cells and cytotoxic T lymphocytes (CTLs) to eliminate tumor cells [29,30]. Additionally, IFN-I enhances antigen presentation and improves the ability of DCs to prime T cells, thus enhancing adaptive anti-tumor immunity.

Beyond immune activation, cGAS-STING signaling directly induces tumor cell apoptosis, targeting genomically unstable cells and reinforcing immune-mediated tumor clearance. Endoplasmic reticulum stress, NLRP3 inflammasome activation, NF-κB pathway, and IFN-I signaling downstream of STING are closely related to cGAS-STING-mediated apoptosis of tumor cells [31,32]. Additionally, acute activation of cGAS-STING signaling induces remodeling of the tumor immune microenvironment to foster an anti-tumor state. cGAS-STING activation can reduce immunosuppressive regulatory T cells (Tregs) in the TME, alleviating their inhibition of immune responses [33]. Additionally, it reprograms tumor-associated macrophages (TAMs) to a pro-inflammatory M1 phenotype, enhancing cytotoxic immune cell infiltration and promoting tumor regression [34,35].

**Figure 2:**
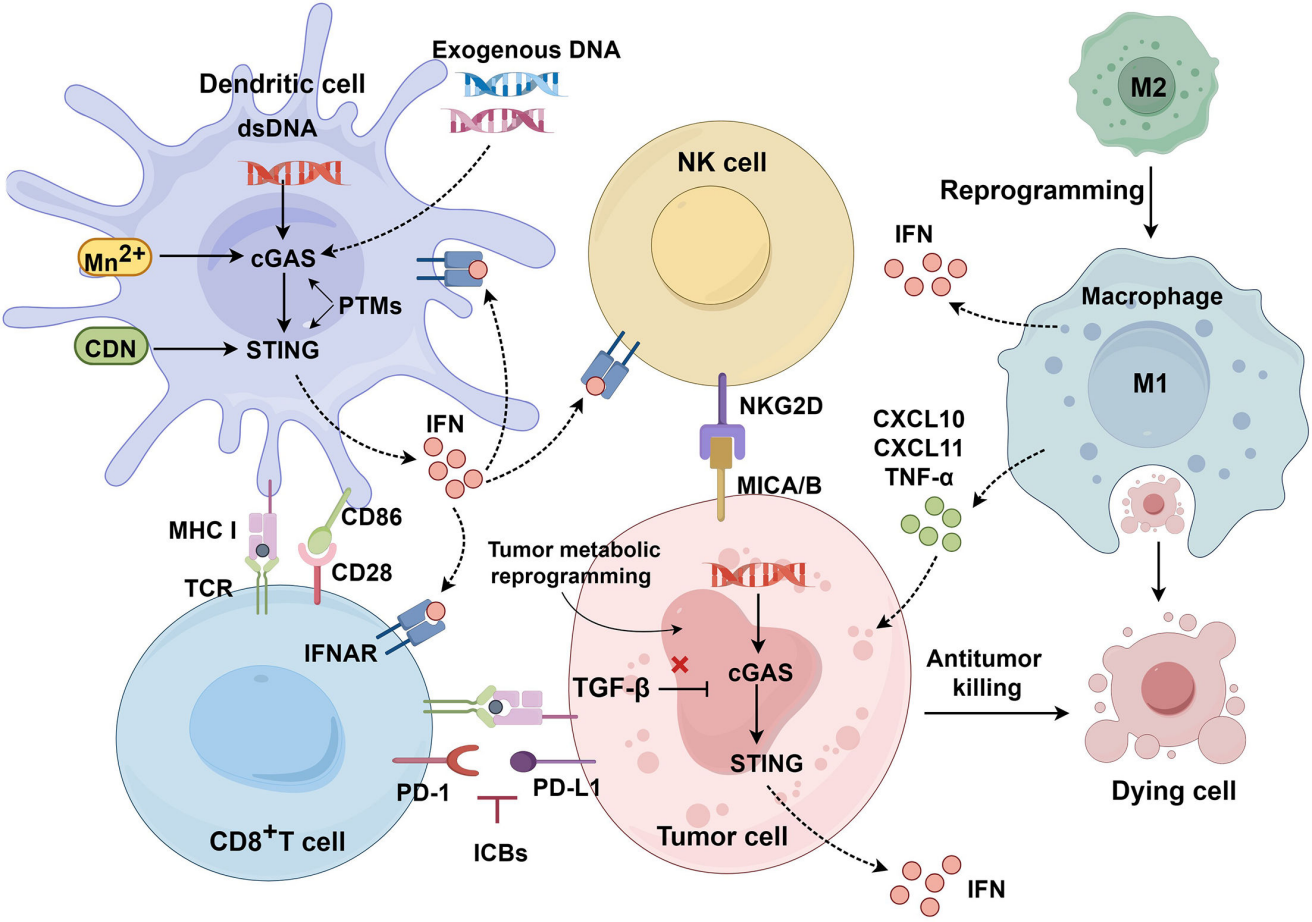
Anti-tumor role of cGAS-STING pathway. Aberrant cytoplasmic DNA activates the cGAS-STING pathway in DCs to induce the production of interferon (IFN) and increase the expression of major histocompatibility complex I (MHC I) and CD86 molecules on the DC surface. Activated DCs promote cross-presentation of tumor antigen to T cells, which further activates T cells and exerts anti-tumor effects. Mass production of IFNs also up-regulates immunosuppressive factors such as PD-L1 on the tumor surface, so combination therapy with synergistic ICB therapy is required. Activation of STING promotes macrophage maturation and migration to the tumor site, exhibiting potent phagocytic activity and increasing levels of CXCL10, CXCL11, and TNF-α. The cGAS-STING signaling pathway also up-regulates the expression of the natural killer group 2 member D (NKG2D) ligand on tumor cells, which binds to NKG2D receptors on NK cells, thus enhancing NK cell-mediated tumor cell killing. And there are three main ways to effectively activate cGAS-STING pathway. Direct activation using STING agonists such as CDNs, or introduction of exogenous DNA into the tumor. The cGAS or STING activity can be regulated by PTMs. The second strategy is to modulate the TME, such as targeting tumor metabolism or suppressive pathways with specific inhibitors. It can also activate cGAS-STING by increasing the activity of immune cells, such as STING agonists in combination with ICBs. Created with Figdraw. CDNs, cyclic dinucleotides; cGAS-STING, cyclic GMP-AMP synthase–stimulator of interferon genes; DCs, dendritic cells; ICBs, immune checkpoint blockades; PTMs, post-translational modifications; TME, tumor microenvironment.

Furthermore, the cGAS-STING pathway supports long-term anti-tumor immune memory by activating DCs, potentially preventing recurrence. These characteristics suggest that the cGAS-STING pathway is a promising investigational target for cancer immunotherapy [36,37] (Figure 2).

### Strategies for cGAS-STING activation

Activation of the cGAS-STING pathway has shown potential as a strategy to enhance anti-tumor immunity. Recent methods can be categorized into the following three main strategies.

#### Direct activation strategies

Direct activation strategies of the cGAS-STING pathway include small-molecule agonists, exogenous DNA delivery, DNA release induction, post-translational modifications (PTMs) regulation, and metal ion enhancement. Synthetic cyclic dinucleotides (CDNs), like 2'3'-cGAMP, directly stimulate STING, inducing strong immune responses and showing promise in preclinical and early clinical studies [38–40]. MIW815 (ADU-S100), a CDN analog, demonstrated immune-stimulating effects. A Phase Ib trial in 106 patients with advanced solid tumors or lymphomas showed that MIW815 combined with spartalizumab-enhanced immune responses, inhibiting tumor growth and spread. Common side effects included fever, injection site pain, and diarrhea [41,42]. Another approach involves introducing exogenous DNA into tumor cells to activate cGAS [43,44]. Inducing tumor cell apoptosis or necrosis provides another method of triggering the cGAS-STING pathway [45]. Additionally, metal ions such as manganese ions (Mn^2+^) have emerged as potent activators of the cGAS-STING pathway. Based on this, we have designed RMPs@Mn^2+^ hydrogel to efficiently amplify the cGAS-STING cascade of antigen-presenting cells (APCs) and promote T-cell priming and infiltration, thus resulting in tumor regression [46]. Collectively, these diverse approaches provide a comprehensive framework for effectively activating the cGAS-STING pathway in cancer immunotherapy.

#### TME modulation

The TME is a complex and dynamic network of cellular and non-cellular components that often suppresses cGAS-STING signaling through immune evasion mechanisms and metabolic aberrations [47,48]. Strategies to modulate the TME aim to reshape its immune landscape and metabolic features to amplify cGAS-STING activation and restore anti-tumor immunity [49]. One approach involves targeting tumor metabolic reprogramming, as tumor cells often exhibit heightened oxidative stress and mitochondrial dysfunction, leading to the release of mitochondrial DNA (mtDNA) into the cytosol [50–52]. Enhancing oxidative stress or disrupting mitochondrial metabolism can promote mtDNA release, thereby boosting cGAS-STING signaling [53,54]. Furthermore, targeting immunosuppressive pathways with specific inhibitors restores the activation potential of the cGAS-STING pathway [55–57].

#### Immune cell function enhancement

DCs, as key mediators of tumor-derived DNA recognition, play a crucial role in initiating immune responses via the cGAS-STING pathway [58]. By utilizing small-molecule drugs to enhance DC function, antigen presentation and downstream STING activation can be significantly amplified [57]. The combination of STING agonists with ICBs has demonstrated the potential to enhance T-cell infiltration and activation, boosting T-cell-mediated tumor eradication [59]. Moreover, reprogramming TAMs into a pro-inflammatory state further strengthens anti-tumor immunity. Together, these immune cell-targeted strategies provide a robust approach to indirectly activating the cGAS-STING pathway and achieving more effective cancer immunotherapy [60].

## Protumor role of cGAS-STING pathway

### Tumor-promoting mechanisms

The cGAS-STING pathway not only supports anti-tumor immunity but can also promote tumors and immune suppression in certain contexts. Chronic activation, especially in high-CIN tumors, may drive inflammation, immune evasion, and metastasis [61–63]. This duality reflects the complexity of the pathway and underscores the need to balance its activation. The following section delves into the protumor effects of chronic cGAS-STING activation.

#### Chronic cGAS-STING activation and immune suppression

Under normal conditions, cGAS-STING activation leads to the production of IFN-I and inflammatory cytokines, enhancing anti-tumor immunity [25]. However, chronic cGAS-STING activation can induce immune suppression through non-classical NF-κB signaling [64]. Persistent production of immune-suppressive cytokines, such as IL-10 and TGF-β, can suppress the function of CTLs and NK cells, which foster the accumulation of Tregs [30]. Additionally, Li et al. reported that the administration of STING agonists can expand IL-35^+^ regulatory B cells (Bregs) in an IRF3-dependent, but type I IFN-independent, manner. These Bregs secrete IL-35, which suppresses NK cell proliferation and attenuates NK-driven anti-tumor immunity. Furthermore, blocking IL-35 in Bregs improves tumor control, highlighting the role of the STING/IRF3/IL-35 axis in shaping an immunosuppressive microenvironment [65].

#### cGAS-STING-mediated immune evasion through PD-L1 up-regulation

One of the key immune evasion mechanisms driven by chronic cGAS-STING activation is the up-regulation of immune checkpoint molecules, particularly PD-L1, which leads to T-cell exhaustion and impaired anti-tumor immunity [66]. Although the exact mechanism remains unclear, current studies suggest that the up-regulation of PD-L1 following cGAS-STING activation is associated with NF-κB pathway and cytokine modulation. NF-κB, particularly via the p65 subunit, induces the expression of COP9 signalosome 5, which is essential for the stabilization of PD-L1 by inhibiting its ubiquitination and degradation [67].

#### Chronic inflammation and protumorigenic TME

Sustained cGAS-STING activation persistently produces pro-inflammatory cytokines, which support immune evasion and promote tumor cell proliferation [68]. Additionally, chronic inflammation leads to the reprogramming of TAMs into a protumor M2 phenotype, which facilitates tumor growth and immune suppression [69]. Furthermore, sustained inflammation stimulates fibrosis in the tumor stroma. Fibroblasts are recruited to deposit extracellular matrix components, creating a fibrotic barrier that impedes immune cell infiltration and supports immune evasion, providing a protective niche for tumor cells to survive [70]. cGAS-STING activation can also promote tumor metastasis in cell-autonomous and non-cell-autonomous manners [71].

### Strategies to overcome the tumor-promoting effects of the cGAS-STING pathway

#### Reducing chronic inflammation in the TME

Chronic cGAS-STING activation up-regulates immune checkpoint molecules, particularly PD-L1 [72]. For tumors with high PD-L1 expression, a dual blockade targeting both PD-1 and PD-L1 combined with STING agonists may reinvigorate T-cell responses and improve treatment efficacy [73,74].

Since chronic inflammation contributes to tumor progression, anti-inflammatory treatments can help reverse the protumor effects of cGAS-STING activation. Non-steroidal anti-inflammatory drugs, such as aspirin, can reduce inflammatory cytokines, which are induced by chronic STING activation [75]. Targeting specific cytokines, e.g., IL-1β, IL-6, and TNF-α, can help alleviate systemic and localized inflammation. Additionally, reprogramming TAMs to an anti-tumor M1 phenotype using CSF-1R inhibitors or anti-CD40 antibodies could further reduce immune suppression [76,77].

#### Inhibition of prolonged cGAS-STING activation

For tumors with high CIN, where cGAS-STING is persistently activated, inhibiting the pathway can prevent tumor progression. Small molecule inhibitors targeting cGAS and STING have been identified. For example, XQ2 specifically binds to cGAS, blocks the binding of dsDNA to cGAS, and inhibits dsDNA-induced cGAS activation. However, XQ2 showed strong cytotoxicity and poor solubility in *in vitro*. G150 and G108 block the ATP/GTP binding pockets of cGAS, preventing downstream signaling. Further studies showed that they had low cytotoxicity and could specifically inhibit dsDNA-activated cGAS activation in human monocyte [78]. Similarly, STING inhibitors like SN-011 and BB-Cl-amidine block STING activation, preventing the inflammatory cascade [79,80]. These inhibitors can reverse the tumor-promoting effects of chronic cGAS-STING activation. In future clinical studies for tumor treatment, these inhibitors may have the effect of improving the inflammatory microenvironment of tumors.

#### Localized STING agonist delivery

Instead of systemic STING agonist therapy, which could exacerbate inflammation, localized delivery of STING agonists can provide a safer and local immune response. The immunosuppressive TME can hinder the function of APCs and T cells. And intratumoral injection of STING agonists triggers local immune activation without causing widespread inflammation [81]. For example, Boudreau et al. have found that intratumoral injections of STING agonists can produce a clinical response to canine glioblastoma [82]. Additionally, using nanoparticles or liposomes to deliver STING agonists ensures targeted delivery to the tumor site, protecting the agonists from degradation and enhancing their concentration in the TME [83]. Song et al. constructed so-called M@P@HA nanoparticles to deliver STING agonists locally to mouse tumors, which can effectively activate innate immunity and cascade activate T cells [84].

#### Monitoring and modulating cGAS-STING activation with biomarkers

Biomarkers can help guide therapy and ensure appropriate modulation of the cGAS-STING pathway [85,86]. Biomarkers for CIN and STING activation may be useful to identify which tumors will benefit from treatment with STING agonists or inhibitors. The key molecules in the cGAS-STING signaling pathway can serve as predictive biomarkers to guide cancer treatment. Phosphorylation of TBK1 and IRF3 is critical for downstream signaling in the cGAS-STING pathway; therefore, pTBK1 and pIRF3 can serve as predictive indicators [87]. The CIN phenotype can be determined by the loss of heterozygosity status to determine whether the patient is a candidate for STING agonist [88].

**Figure 3:**
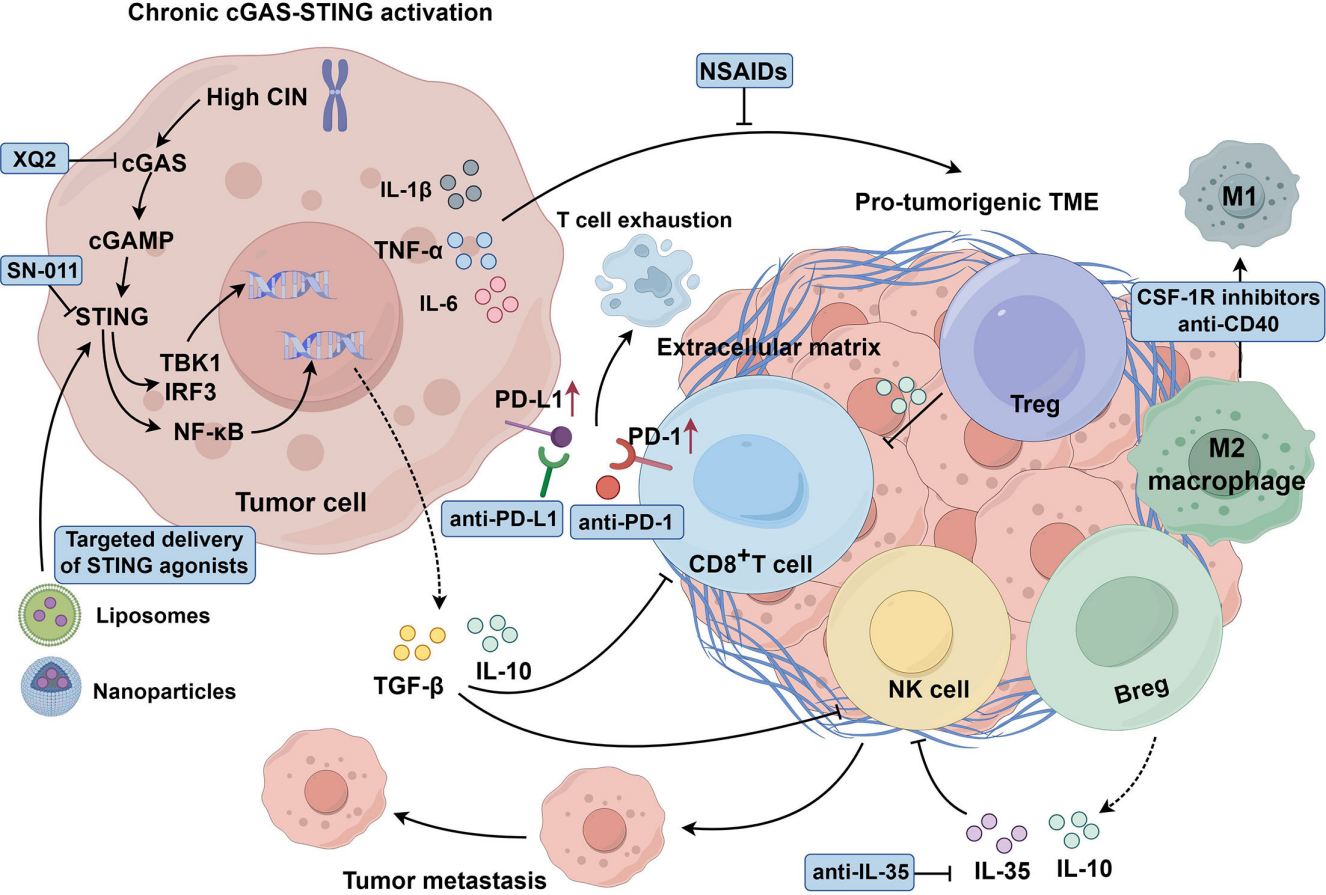
Protumor role of cGAS-STING pathway. In high CIN tumors, persistent cGAS-STING activation drives inflammation-driven carcinogenesis. STING activation in tumor cells promotes immune-suppressive cytokines production, such as IL-10 and TGF-β, inhibiting T and NK cell function while promoting Treg accumulation. It also triggers pro-inflammatory cytokines, such as IL-1β, TNF-α, and IL-6, supporting immune evasion and tumor proliferation. Additionally, aberrant activation up-regulates PD-L1 in tumors and PD-1 in T cells, enhancing immune escape and metastasis. STING activation promotes the proliferation of Breg and secreted inflammatory factors such as IL-35 and IL-10, further inhibiting NK cells. Chronic inflammation reprograms TAMs into the protumor M2 phenotype, fostering tumor growth and immune suppression. Fibroblasts deposit ECM components to form a barrier that impedes immune cell infiltration and supports immune evasion. To counteract these effects, strategies include combining STING agonists with immune checkpoint blockades (ICBs), using NSAIDs to reduce inflammation, reprogramming TAMs from M2 to M1 with CSF-1R inhibitors or anti-CD40 antibodies, and employing small-molecule inhibitors to regulate cGAS-STING activity. Nanoparticle or liposome-based STING agonist delivery can enhance tumor targeting and efficacy. Created with Figdraw. cGAS-STING, cyclic GMP-AMP synthase–stimulator of interferon genes; CIN, chromosomal instability; ECM, extracellular matrix; NSAIDs, non-steroidal anti-inflammatory drugs.

These approaches could significantly enhance personalized cancer immunotherapy, improving efficacy while minimizing tumor progression (Figure 3).

## Current challenges and solutions for cGAS-STING targeted therapies in clinical applications

### Key challenges in the clinical application

#### Immune-related adverse events

The most significant challenges in targeting the cGAS-STING pathway are immune-related adverse events (irAEs) [89]. Activation of STING can lead to the production of IFN-I and other inflammatory cytokines, which can enhance anti-tumor immunity and cause systemic inflammation, leading to side effects such as autoimmune responses, fatigue, and tissue damage [90]. Inflammatory diseases like lupus or rheumatoid arthritis are linked to dysregulated STING activation, which underscores the need for precise control over the cGAS-STING pathway activation [91].

#### Tumor heterogeneity and resistance mechanisms

Tumor heterogeneity affects the efficacy of cGAS-STING-targeted therapies. Both genetic mutations and epigenetic alterations contribute to variations in STING signaling, leading to differential responses across cancer types [92]. In melanoma, cGAS-STING signaling is often impaired due to promoter methylation of STING, which limits its ability to trigger effective IFN-I responses. DNA methylation inhibitors can restore STING function, enhancing antigenicity and tumor immunogenicity [93]. Moreover, in glioma, STING silencing mediated by hypermethylation of CpG site cg16983159 located on STING promoter may reduce sensitivity to cGAMP but can be restored using DNA methyltransferase inhibitors [94]. These insights highlight the complexity of cGAS-STING signaling across different cancer types and reinforce the need for patient stratification strategies, including genomic and epigenetic profiling, to optimize cGAS-STING-targeted therapies.

#### Delivery and bioavailability of STING agonists

Developing effective and safe delivery systems for STING agonists remains a considerable challenge. Many STING agonists are associated with poor bioavailability, rapid degradation, and inefficient cellular uptake [41,95]. The systemic administration of STING agonists may lead to insufficient concentrations at the tumor site, limiting their therapeutic efficacy. Moreover, local delivery systems may be needed to maximize the tumor-targeting effect and minimize systemic toxicity.

### Potential solutions for overcoming challenges in cGAS-STING targeted therapies

#### Controllable STING agonists development and biomarker-driven patient selection

To address toxicity concerns, researchers are focusing on developing more selective STING agonists that can modulate the pathway in a controlled manner [96]. These agonists aim to activate STING only in the presence of specific tumor-associated signals, thereby reducing the risk of systemic inflammation [97]. Jie et al. developed a non-nucleotide small-molecule agonist, NVS-STG2, which acts specifically on human STING proteins and activates STING-mediated immunity in a dose-dependent manner [98,99].

To minimize irAEs and enhance therapeutic efficacy, the development of biomarkers for patient selection is crucial [100]. Identifying patients who are most likely to benefit from STING-targeted therapies, based on their tumor characteristics, can improve clinical outcomes and reduce unnecessary side effects. Liquid biopsies and molecular profiling may help to identify biomarkers that predict response to STING agonists, enabling personalized treatment strategies [101].

#### Combination therapies to overcome resistance

To combat tumor heterogeneity and resistance, combination therapies are being explored [102]. Combining STING agonists with other immune-modulating therapies, such as checkpoint inhibitors, may enhance the efficacy of STING activation and help overcome tumor resistance mechanisms [103,104]. Additionally, the use of STING agonists in combination with conventional therapies such as chemotherapy or radiotherapy could potentially sensitize tumors to immune-mediated killing. Dong et al. found that anlotinib could further increase radiotherapy-stimulated CD8^+^ T-cell infiltration and activation by triggering the cGAS/STING pathway [105].

#### Improved drug delivery systems

Advances in nanotechnology and targeted drug delivery are providing promising solutions for the efficient delivery of STING agonists [106]. Lipid-based nanoparticles, polymeric micelles, and exosome-based carriers are under investigation that may enhance the bioavailability and stability of STING agonists [96,107]. Dosta et al. developed poly (β-amino ester) nanoparticles (CDN-NP) with a cathepsin-sensitive linker. This material accumulated efficiently in the mouse spleen and was internalized by APCs such as DC. CDN-NP ingested by tumor cells would be released again to activate immune cells, thereby activating cGAS-STING signaling and inducing a strong anti-tumor immune response [108].

**Figure 4:**
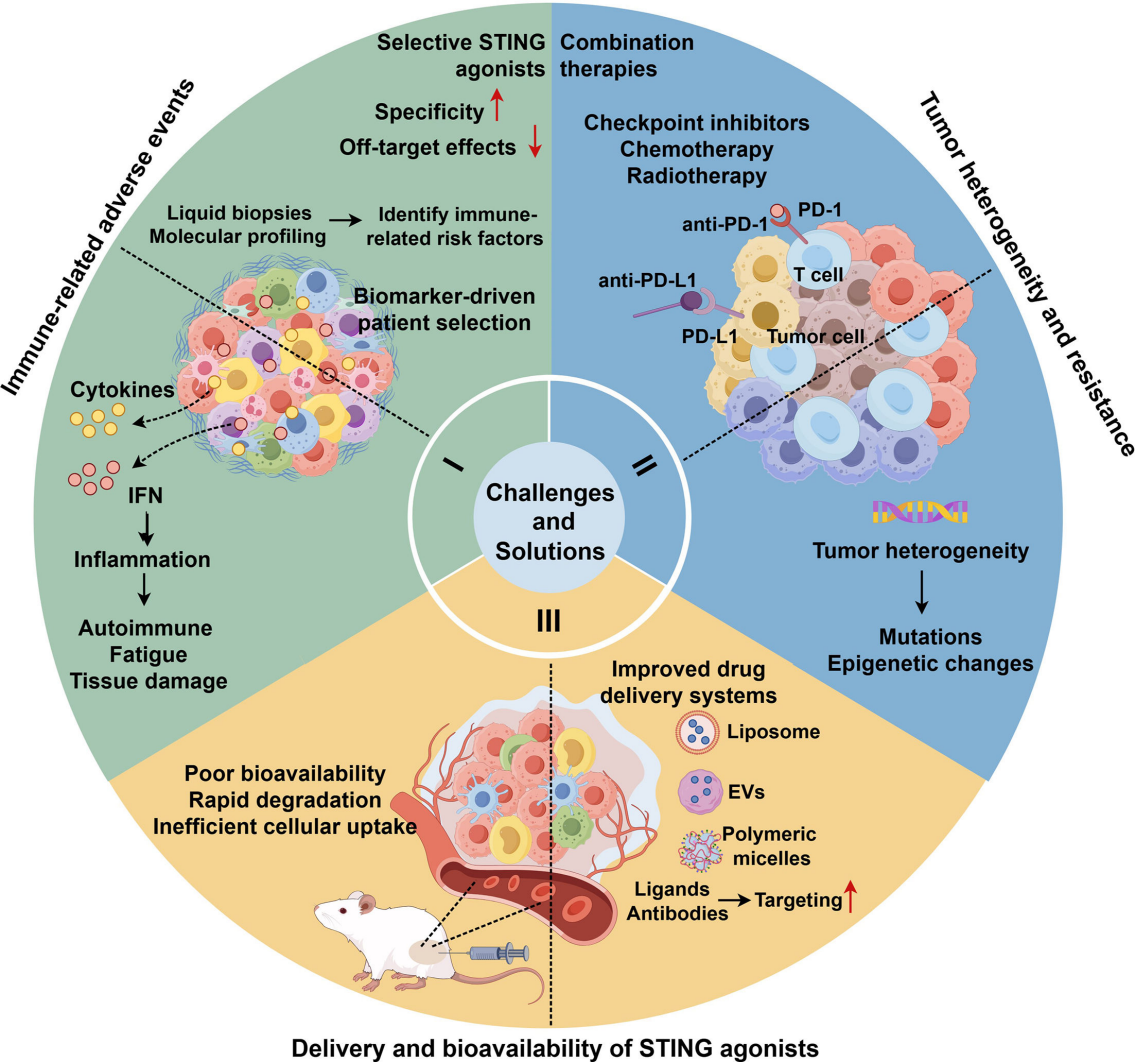
Challenges and solutions for future research on the cGAS-STING signaling pathway in tumor treatment. Part I: The use of STING agonists has the problem of poor targeting, which can induce systemic inflammatory responses, and cause damage to the cells. Developing new STING agonists with better specificity can improve their pharmacokinetic properties and reduce off-target effects. Identification of biomarkers enables STING-targeted therapies to be more refined and targeted. Distinguish which patients are better candidates for targeted STING therapy to make the treatment more effective and safer. Part II: There is genetic and phenotypic heterogeneity in tumor cells, and a single treatment method cannot solve the treatment resistance caused by tumor heterogeneity, and it is necessary to combine different treatment strategies to improve the treatment effect. Part III: The bioavailability of STING agonists is poor. It is rapidly degraded in the body and cannot be efficiently taken up by cells. The use of extracellular vesicles, organic polymers, and other materials to deliver agonists can effectively solve this problem. Created with Figdraw. STING, stimulator of interferon genes.

cGAS-STING-targeted therapies hold significant potential in oncology but are limited by challenges such as toxicity, tumor resistance, delivery barriers, and irAEs [109]. Advancements in selective agonists, combination strategies, optimized delivery systems, and biomarker-guided patient selection are essential to improve their clinical efficacy and safety (Figure 4).

## Conclusion and future outlook

cGAS-STING signaling pathway offers immense potential for revolutionizing cancer immunotherapy. However, significant challenges remain in translating preclinical findings into effective clinical treatments, including heterogeneity, delivery systems, immune suppression in solid tumors, which must be overcome before the full potential of cGAS-STING-targeted therapies can be realized. The future of cGAS-STING-targeted therapies lies in personalized medicine and combination strategies to enhance immune activation while minimizing adverse effects.

Recent advances in STING agonist, such as non-nucleotide small molecules like NVS-STG2, show promise for improving specificity and efficacy, particularly in tumors with high mutational burdens [98]. Additionally, research should optimize combination therapies, like STING agonists with ICBs, CAR-T, oncolytic viruses, and photothermal therapy, to overcome single-agent limitations and enhance anti-tumor responses.

The identification and application of predictive biomarkers are critical to improve patient selection and treatment outcomes. Promising candidates include cGAMP levels, IRF3 activation, and PD-L1 expression, which could guide therapeutic decisions and monitor response to STING-based therapies. Advances in liquid biopsy techniques are enabling real-time monitoring of these biomarkers, paving the way for dynamic and personalized treatment adjustments.

By focusing on the development of novel STING agonists, strategic combination therapies, and actionable biomarkers, future research can provide clearer and more targeted solutions for overcoming current limitations in cGAS-STING-targeted cancer immunotherapy.

**Summary**

- The cyclic GMP-AMP synthase–stimulator of interferon genes (cGAS-STING) pathway activation enhances immune responses and reshapes the tumor microenvironment to support immune activation.
- Chronic cGAS-STING pathway activation can lead to immune suppression.
- Effective cGAS-STING targeting requires balancing immune activation while minimizing protumor effects of chronic activation.
- Further researches are essential to unravel the dual roles of cGAS-STING to maximize its clinical utility in cancer immunotherapy.

## Abbreviations

APCs: antigen-presenting cells
Bregs: regulatory B cells
CDNs: cyclic dinucleotides
CIN: chromosomal instability
CTLs: cytotoxic T lymphocytes
DCs: dendritic cells
ECM: extracellular matrix
ICBs: immune checkpoint blockades
IFN-I: type I interferons
IKK: inhibitor of κB kinase
MHC I: major histocompatibility complex I
Mn^2+^: manganese ions
NK: natural killer
NKG2D: natural killer group 2 member D
NSAIDs: non-steroidal anti-inflammatory drugs
PTMs: post-translational modifications
STING: stimulator of interferon genes
TAMs: tumor-associated macrophages
TBK1: TANK-binding kinase 1
TGF-β: transforming growth factor-beta
TME: tumor microenvironment
Tregs: regulatory T cells
cGAMP: cyclic GMP-AMP
cGAS: cyclic GMP-AMP synthase
dsDNA: double-stranded DNA
irAEs: immune-related adverse events
irAEs: immune-related adverse events
mtDNA: mitochondrial DNA
